# Effects of Therapy on Urine Neutrophil Gelatinase-Associated Lipocalin in Nondiabetic Glomerular Diseases with Proteinuria

**DOI:** 10.1155/2016/4904502

**Published:** 2016-07-25

**Authors:** Amnuay Sirisopha, Somlak Vanavanan, Anchalee Chittamma, Bunyong Phakdeekitcharoen, Ammarin Thakkinstian, Amornpan Lertrit, Nuankanya Sathirapongsasuti, Chagriya Kitiyakara

**Affiliations:** ^1^Department of Medicine, Faculty of Medicine Ramathibodi Hospital, Mahidol University, Bangkok 10400, Thailand; ^2^Department of Pathology, Faculty of Medicine Ramathibodi Hospital, Mahidol University, Bangkok 10400, Thailand; ^3^Section for Clinical Epidemiology and Biostatistics, Faculty of Medicine Ramathibodi Hospital, Mahidol University, Bangkok 10400, Thailand; ^4^Graduate Program in Translational Medicine, Research Center, Faculty of Medicine Ramathibodi Hospital, Mahidol University, Bangkok 10400, Thailand

## Abstract

Urine neutrophil gelatinase-associated lipocalin (NGAL) is widely used as a biomarker for acute kidney injury. Cross-sectional studies have shown that NGAL may be elevated in glomerular diseases, but there is limited information on the value of NGAL in predicting treatment response or on the changes of NGAL levels after therapy. We prospectively evaluated the effects of therapy on NGAL in nondiabetic glomerular diseases. Urine NGAL was collected at biopsy and follow-up at 12 months. At baseline, NGAL in glomerular disease patients (*n* = 43) correlated with proteinuria, but not with glomerular filtration rate (GFR). After therapy with renin-angiotensin blockers and/or immune modulating agents, change of NGAL correlated with change of proteinuria, but not with change of GFR. NGAL at baseline was not different between patients in complete remission (CR) at follow-up compared to those not in remission (NR). Compared to baseline, NGAL at follow-up decreased in CR (*n* = 10), but not in NR. Change of NGAL was greater in CR than NR. In conclusion, the change of urine NGAL correlated with the change of proteinuria. Baseline NGAL was not a predictor of complete remission. Future studies will be necessary to determine the role of NGAL as a predictor of long term outcome in proteinuric glomerular diseases.

## 1. Background

Glomerular disease consists of a group of disorders that together constitutes one of the leading causes of end-stage renal disease (ESRD) worldwide [1]. Once established, proteinuric glomerular disease causes activation of pathogenic processes leading to chronic tubular injury, fibrosis with subsequent nephron loss, and progressive decline in renal function [2]. Proteinuria is an important direct mediator of tubular epithelial cell injury and is a strong predictor of renal disease progression [3]. Reducing proteinuria with immune modulating therapy or renin-angiotensin system blockers has been shown to improve outcome in diverse types of glomerular diseases. However, response to therapy is variable and progressive nephron loss could still occur at dissimilar rates. A noninvasive biomarker that could predict response to treatment or prognosis would be useful in the management of glomerular diseases.

Neutrophil gelatinase-associated lipocalin (NGAL) is a small 25-kDa protein of the lipocalin family. After acute kidney injury, intrarenal NGAL is markedly upregulated [4] and NGAL is excreted in the urine in parallel with the severity of tubular injury. Urine NGAL is now widely used as a biomarker for acute kidney injury (AKI). Recently, urine NGAL has also been shown to be elevated in patients with chronic kidney diseases (CKD) of different etiologies. Cross-sectional studies found that urine NGAL was higher in patients with glomerulonephritis [5], diabetic nephropathy [6], and adult polycystic kidney disease [7] compared with healthy controls. Prospective studies suggest that urine NGAL, measured once at baseline, may be a useful predictor for loss of renal function in CKD patients with low level protein excretion [8] or the general population [9]. While several investigators have proposed that NGAL might be a useful biomarker in CKD subjects without significant proteinuria, there is still limited information on the prognostic role of NGAL in proteinuric glomerular diseases. Preliminary studies have shown that baseline NGAL levels may correlate with adverse prognosis in adults with membranous nephropathy and in nephrotic children [5, 10]. However, there are few prospective data on the value of NGAL for predicting therapeutic response in common glomerular diseases. Moreover, previous studies have evaluated NGAL only once at baseline and the relationship between changes of urine NGAL over time in response to treatment has not been fully studied. This information is important if NGAL is to be considered as a biomarker to monitor disease progression. In this study, we will test the hypothesis that NGAL levels can predict medium term response to therapy and that treatment of glomerular diseases will decrease urine NGAL and assess the relationship between changes in urine NGAL excretion with changes of clinical parameters in proteinuric patients with common biopsy-proven, nondiabetic glomerular diseases.

## 2. Materials and Methods

### 2.1. Patients and Baseline Data

This single center, prospective cohort study enrolled adult patients with glomerular diseases referred to the nephrology outpatient clinic of Ramathibodi Hospital during 2013 to 2015. All procedures performed in studies involving human participants were in accordance the 1964 Helsinki Declaration and its later amendments and approved by the Ethics Committee of the Ramathibodi Hospital. Written informed consent was obtained.

Inclusion criteria were biopsy-proven glomerulonephritis and the presence of proteinuria (urine protein creatinine ratio > 0.50 g/g creatinine) and a stable renal function. Patients with kidney transplant, diabetic nephropathy, active infections, or other severe intercurrent illnesses were excluded from the study. The patients' history and clinical examination data were carefully recorded. Patients were given standard treatment including renin-angiotensin system blockade (ACEi-angiotensin converting enzyme inhibitors or ARB-angiotensin receptor blockers) and/or immunosuppressive agents (corticosteroids or other immune modulating drugs or both) according to standard guidelines [11].

Urine samples were also collected from healthy volunteers and from patients with acute kidney injury (AKI). Healthy controls were recruited from volunteers with no chronic illnesses including hypertension or kidney diseases after detailed history taking, physical examination, and routine blood tests including urinalysis and serum creatinine. AKI controls were recruited from hospitalized patients without glomerular diseases who developed acute kidney injury due to nephrotoxic or ischemic insults (defined by KDIGO guideline 2012) [12].

### 2.2. Pathologic Studies

Kidney biopsies were fixed in histological fixative (Glyo-Fixx, Thermo scientific, USA) and paraffin embedded, and sections (2 *μ*m) were processed for light microscopy (hematoxylin and eosin, periodic acid-Schiff, Masson's trichrome, and silver staining), immunofluorescence, and electronmicroscopy and evaluated by a nephropathologist blinded to the laboratory and NGAL data. Glomerular diseases were classified according standard criteria [11].* Tubular injury* was present if there were apical blebs, attenuation of brush border epithelium, sloughed epithelium, or evidence of tubular regeneration. The severity of interstitial fibrosis and tubular atrophy (IFTA) was assessed semiquantitatively as a proportion relative to the total section area as follows: none, <5%; mild, 5–25%; moderate, 26–50%; and severe, >50%.

### 2.3. Laboratory Measurements and Definitions

Baseline blood and second void urine samples were collected on the day of the biopsy and follow-up samples were collected 12 months later.

Common biochemical parameters were measured in a laboratory in compliance with ISO 15189. Creatinine was measured by enzymatic method. Urine samples were centrifuged at 3000 rpm for 10 minutes at 4°C and the supernatant was sent for analysis for NGAL using a chemiluminescent microparticle immunoassay (CMIA) kit (The ARCHITECT Urine NGAL assay). Coefficient of variation at the low (20.2 ng/mL), medium (196.7 ng/mL), and high (1174.4 ng/mL) urine NGAL levels was 4.4%, 3.0%, and 2.2% for intra-assay variation, respectively, while that for the interassay was 2.1%, 1.7%, and 1.4%, respectively. Using the same aliquot, urine protein was measured by modified pyrogallol red-molybdate method and urine creatinine by enzymatic method on the Dimension ExL analyzer (Siemens Healthcare Diagnostics, Newark, DE, USA).

Glomerular filtration rate (GFR in mL/min/1.73 m^2^) was calculated by using the CKD-EPI equation [13]. Urine protein was reported as urine protein creatinine ratio (UPCR in mg/mgCr).

*Nephrotic range proteinuria* was defined as UPCR more than 2000 mg/mg [11].* Low GFR* was defined as GFR < 60 [14].* Complete remission* was defined as UPCR < 0.3 at the follow-up period [11]. Subjects not in complete remission* (not in remission)* were further subclassified* as partial remission*, defined as 50% or greater reductions in proteinuria, or* resistant disease*, defined as less than 50% reduction in proteinuria or greater than 30 mL/min/1.73 m^2^ decrease in GFR at follow-up.

### 2.4. Statistical Analysis

Data are presented as mean ± standard deviation, median (range), or percentage (frequency), as appropriate. Change in parameters was calculated by subtracting follow-up values from baseline such that positive values represent an increase. These within-individual changes were compared by Wilcoxon test, and changes between groups were compared using independent *t*-test if data were normally distributed; otherwise Mann-Whitney *U* test or Kruskal-Wallis test was applied. Chi-square test was applied to compare distributions for categorical variables. In addition, Spearman's rank correlation was used to assess the correlations between urine NGAL and other variables. A mixed-effect logistic regression was used to assess correlation between GFR group and other variables. All analyses were performed using STATA version 14. All results were considered significant if *p* was <0.05.

## 3. Results

### 3.1. Patient Characteristics

A total of 43 patients of glomerular disease were enrolled (IgA nephropathy (*n* = 10), lupus nephritis class III/IV (*n* = 9), focal segmental glomerulosclerosis (*n* = 7), minimal change disease (*n* = 8), membranous nephropathy (*n* = 5), and others (*n* = 4)). The main baseline characteristics of the study cohort are summarized in Table 1. Mean age of patients was 45 ± 17 years and 34.9% were male. Thirty-eight patients (88.3%) received ACEI or ARB therapy. Twenty-five (58%) patients received immune modulating agents; 7 (16.3%) received only corticosteroids and 18 (41.9%) received a combinations of corticosteroids and immunosuppressive agents.

### 3.2. Proteinuria, GFR, and NGAL at Baseline and Follow-Up

Overall, protein excretion tended to decrease from baseline (*t* = 1) to follow-up (*t* = 2) (UPCR_1_, 2.17 (0.09–9.23) versus UPCR_2_, 0.67 (0.06–16.96) g/g, *p* = 0.12). GFR did not change significantly (GFR_1_, 66 (12–143) versus GFR_2_, 71 (12–140) mL/min/1.73 m^2^, *p* = 0.76) and neither did NGAL (NGAL_1_, 26.1 (2.3–213.0) versus NGAL_2_, 20.8 (0.5–359.7) ng/mL, *p* = 0.96).

Median NGAL in glomerular disease patients at baseline was about 6-fold higher than healthy subjects (NGAL_1_ GN: 26.1 (2.3–213.0) versus healthy, 4.4 (3.1–10.6), *n* = 10, *p* < 0.001), and about 12-fold lower than AKI controls (NGAL_1_ GN: 26.1 (2.3–213.0) versus AKI, 302.6 (85.9–4808), *n* = 19, *p* < 0.001).

### 3.3. Relationship between Urine NGAL with Proteinuria and GFR at Baseline

**Figure 1:**
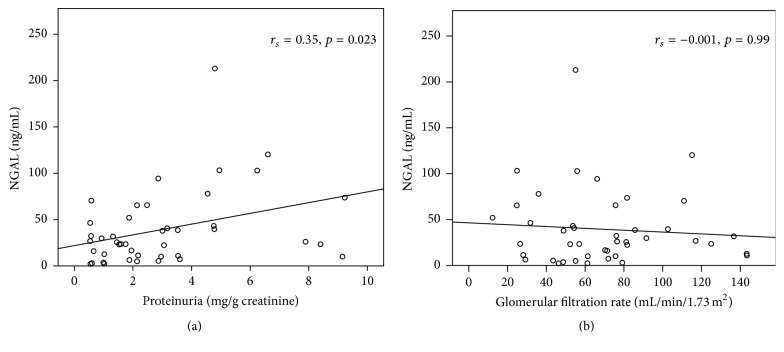
Relationship between NGAL and other laboratory parameters at baseline. (a) Proteinuria at baseline and (b) glomerular filtration rate at baseline (*n* = 43).

Overall, baseline NGAL_1_ (Figure 1(a)) correlated significantly with baseline UPCR_1_ (*r*_*s*_ = 0.346, *p* = 0.023). Twenty-four patients (55.8%) had nephrotic range proteinuria. As expected, nephrotic subjects had higher degrees of proteinuria (UPCR_1_:* nephrotic*, 3.57 (2.14–9.23) versus* subnephrotic*, 1.02, (0.54–1.95, *p* < 0.001)), but GFR was not different (GFR_1_:* nephrotic*, 59 (25–143) versus* subnephrotic*, 71 (12–143), *p* = 0.56). NGAL was higher in nephritic subjects (NGAL_1_:* nephrotic*, 39.2 (5.0–213.0) versus* subnephrotic*, 23.5 (2.3–70.4), *p* < 0.042).

Overall, baseline NGAL did not correlate with baseline GFR (Figure 1(b)). Nineteen patients (44.1%) had* Low GFR* (GFR < 60) with median GFR_1_ of 46.3 (12–57). Baseline GFR in those with preserved GFR (≥60) was 81 (61–143). Baseline proteinuria (UPCR_1_;* low GFR*, 2.18 (0.54–8.40)* versus preserved GFR*, 2.22 (0.54–9.23), *p* = 0.56) and baseline NGAL (NGAL_1_:* Low GFR*, 37.9 (2.3–213.1) versus* preserved GFR*, 25.8 (2.4–120.3), *p* = 0.63) were similar between the two GFR groups.

### 3.4. Relationship between Change of Urine NGAL and Change of Proteinuria or GFR

**Figure 2:**
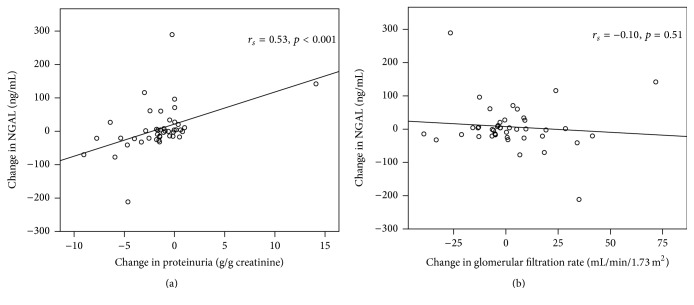
Relationship between change in NGAL and change in other laboratory parameters. (a) Change in proteinuria and (b) change in glomerular filtration rate (*n* = 43) from baseline to follow-up.

From baseline to follow-up, the change of proteinuria (ΔUPCR_2-1_) was −1.38 (−9.03–14.1) g/g Cr, change of GFR (ΔGFR_2-1_) was −0.5 (−39.2–71.7) mL/min/1.73 m^2^, and change of NGAL (ΔNGAL_2-1_) was −0.300 (−211.4–289.3) ng/mL. ΔNGAL_2-1_ significantly correlated with ΔUPCR_2-1_ (*r*_*s*_ = 0.530, *p* < 0.001), but not with ΔGFR_2-1_ (Figure 2).

### 3.5. Relationship between Urine NGAL and Renal Histopathology

To explore the relationship between acute tubular injury and NGAL levels, we divided patients into* tubular injury *(*n* = 32) and* no injury* (*n* = 11) groups according renal histology findings. GFR_1_ at baseline and GFR_2_ at follow-up were higher in* tubular injury*, but there were no differences in proteinuria. There was considerable overlap in baseline NGAL such that there was no statistical difference between the 2 groups. (NGAL_1_:* no injury*, 26.8 (2.4–73.4) versus* tubular injury*, 26.1 (2.3–213.1) ng/mL, *p* = 0.92). No differences were observed in ΔGFR_2-1_, ΔUPCR_2-1_, and ΔNGAL_2-1_ between the 2 groups. It is worth noting that only patients with features of tubular injury (*n* = 5) had baseline NGAL above 85 ng/mL (the lowest level in nonglomerular AKI controls). All five patients had nephrotic syndrome and 4 of these patients had serum albumin less than 2.5 g/dL.

To explore relationship between NGAL and chronic tubulointerstitial changes, subjects were divided into 2 groups according to the severity of interstitial fibrosis and tubular atrophy (IFTA):* none to mild* (*n* = 36) and* moderate to severe* (*n* = 7). GFR at baseline and at follow-up were lower in* moderate to severe* IFTA, but there were no differences in proteinuria (*data not shown*). NGAL tended to be higher in* moderate to severe* IFTA at baseline (NGAL_1_:* none to mild*, 24.6 (2.3–213.0) versus* moderate to severe*, 46.4 (5.4–103.2) ng/mL, *p* = 0.19) but were similar at follow-up (NGAL_2_:* none to mild*, 18.4 (0.5–359.7) versus* moderate to severe*, 25.6 (9.1–80.9) ng/mL, *p* = 0.63). Reduction in NGAL was greater in* moderate to severe* IFTA (ΔNGAL_2-1_:* none to mild*, −2.8 (−211.4–289.3) versus* moderate to severe*, −15.0 (−77.3–3.7) ng/mL, *p* = 0.046). No differences were observed in ΔGFR_2-1_ or ΔUPCR_2-1_ between the 2 groups.

### 3.6. NGAL in Patients with or without Complete Remission

At follow-up, 10 patients (23.2%) were* incomplete remission* (CR) (Table 1). CR was more likely in those who received immune modulating drugs compared to those not in remission (NR). In CR patients, the pathologies were lupus nephritis (*n* = 2), minimal change disease (*n* = 5), IgA nephropathy (*n* = 1), and focal segmental glomerulosclerosis (*n* = 2). Four patients were treated with prednisolone, four had prednisolone and immunosuppressive agents (azathioprine, cyclophosphamide, or mycophenolate mofetil), and 2 had ACEi or ARB without immune modulating agents. All patients received ACEi or ARB except one patient with minimal change disease who had prednisolone alone.

**Figure 3:**
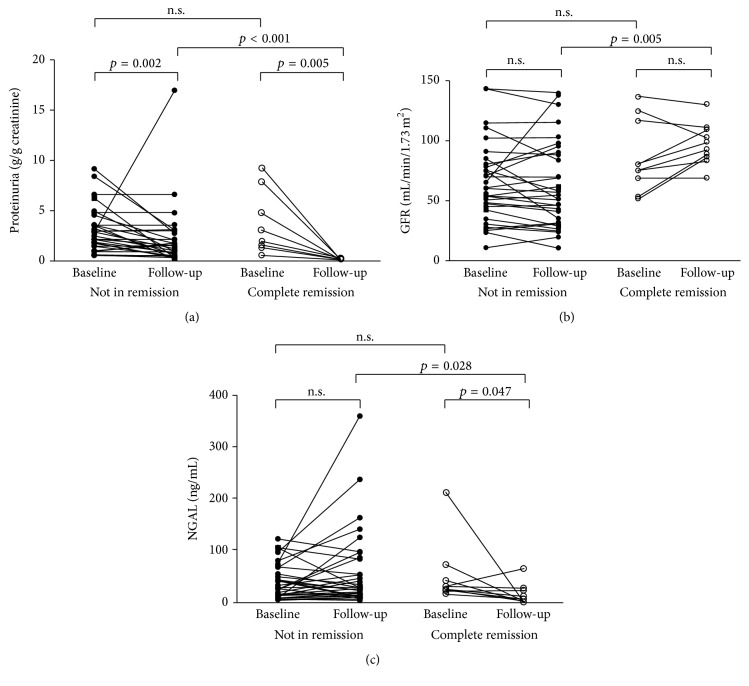
Laboratory parameters according to remission status at follow-up. (a) Proteinuria, (b) glomerular filtration rate, and (c) NGAL at baseline and follow-up. Complete remission (*n* = 10); not in remission (*n* = 33).

Baseline protein (UPCR_1_) was not significantly different between patients who were in CR compared to NR (Figure 3(a)). UPCR decreased significantly at follow-up in both groups (CR: UPCR_1_, 2.51 (0.54–9.23) versus UPCR_2_, 0.15 (0.06–0.26), *p* = 0.005, and NR: UPCR_1_, 2.18 (0.54–9.15), versus UPCR_2_, 1.22 (0.23–16.96), *p* = 0.002). As expected, protein levels at follow-up (UPCR_2_) were lower in CR group compared to NR (*p* < 0.001). Change of proteinuria was greater in those with CR (ΔUPCR_2-1_: CR, −2.28 (−9.03–−0.48), versus NR, −1.05 (−6.40–14.10), *p* = 0.031).

There was no difference in baseline GFR_1_ (Figure 3(b)) between CR versus NR (*p* = 0.28). In NR group, GFR did not change at follow-up (NR: GFR_1_, 57 (12–143), versus GFR_2_, 56 (12–140), *p* = 0.48). In CR group, GFR tended to increase (CR: GFR_1_, 79 (54–137), versus GFR_2_, 97 (71–131), *p* = 0.11). Change in GFR tended to be greater in CR, but this was not significant (ΔGFR_2-1_: CR, +13 (−21–35), versus NR −3 (−39–72), *p* = 0.11). GFR_2_ at follow-up was higher in CR compared to NR (*p* = 0.005).

**Figure 4:**
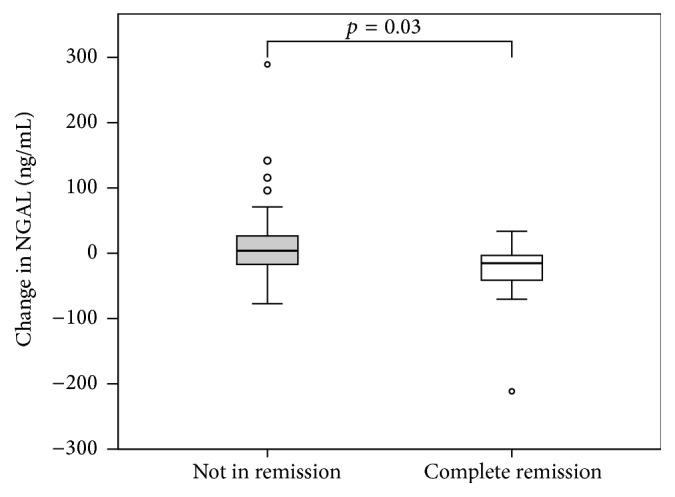
Change in NGAL (ng/mL) levels according to remission status at follow-up. Change in urine NGAL levels from baseline to follow-up between complete remission (*n* = 10) versus not in remission (*n* = 33).

Median NGAL at baseline (Figure 3(c)) were similar between patients with or without remission (*p* = 0.286). Compared to baseline values, NGAL decreased in CR subjects (CR: NGAL_1_, 29.3 (16.7–213.2), versus NGAL_2_, 7.4 (1.6–66.1) ng/mL, *p* = 0.047), but not in patients without remission (NR: NGAL_1_, 23.5 (2.3–120.3), versus NGAL_2_, 25.2 (0.5–359.7) ng/mL, *p* = 0.31). At follow-up, NGAL_2_ was lower in CR compared to NR (*p* = 0.028). Of note, the follow-up level of NGAL_2_ in CR was comparable to those of healthy subjects (*p* = 0.393). The reduction in NGAL was greater in CR compared to NR (ΔNGAL_2-1_: CR, −15.150 (−211.4–33.7), versus NR, 3.9 (−77.3–289.3) ng/mL, *p* = 0.033) (Figure 4).

A simple logistic regression showed that baseline urine NGAL was not a predictor of the remission status (*data not shown*). For all analyses, using log transformed NGAL or adjusting NGAL with urine creatinine concentrations (NGAL/Cr) produced similar results to NGAL alone (*data not shown*).

### 3.7. Partial Remission and Resistant Disease

*Not in remission* subjects (see Supplementary Table  1 in Supplementary Material available online at http://dx.doi.org/10.1155/2016/4904502) were further subclassified into* partial remission* (*n* = 17) and* resistant disease* (*n* = 16). Compared to* resistant disease* (resistant),* partial remission* (PR) had similar proteinuria at baseline, but lower proteinuria and greater reduction in proteinuria at follow-up (UPCR_2_: PR, 0.80 (0.31–13.04), versus resistant, 1.62 (0.34–16.96) g/g, *p* = 0.023; ΔUPCR_2-1_, −2.12 (−6.44–−0.93), versus 0.01 (−1.78–14.1) g/g, *p* < 0.001). GFR and NGAL at baseline or follow-up and ΔNGAL_2-1_ or ΔGFR_2-1_ were similar.

When PR subjects were compared to CR, there were no differences in proteinuria at baseline, but CR patients had lower proteinuria level and greater reduction in proteinuria at follow-up (UPCR_2_: CR, 00.15 (0.06–0.26), versus PR, 0.80 (0.31–3.04) g/g creatinine, *p* < 0.001; ΔUPCR_2-1_, CR −2.28 (−9.03–−0.48), versus PR, −2.12 (−6.44–−0.93) g/g creatinine, *p* < 0.001). GFR at baseline and at follow-up or change in GFR were similar. Although baseline and follow-up NGAL levels were not different, the reduction in NGAL was greater in CR than PR (ΔNGAL_2-1_: CR, −15.2 (−211.3–33.7), versus PR, 2.2 (−77.2–116.1) ng/mL, *p* = 0.046).

## 4. Discussion

Although urine NGAL has long been studied for its usefulness in acute kidney injury, few studies have evaluated the changes of urine NGAL over time in CKD. This study examined prospectively the effects of therapy on urine NGAL levels and the relationship of the change of NGAL with other clinical parameters in common glomerular diseases. The novel aspects of this study are that baseline NGAL level was not predictive of response to therapy and that there was a strong relationship between proteinuria and NGAL at baseline and at follow-up. Patients who were in complete remission with normal protein excretion had reduced NGAL at follow-up with levels comparable to healthy subjects, whereas NGAL levels in patients who were not in remission remained elevated. Changes in NGAL excretion correlated with changes in proteinuria, but not with changes in GFR.

In contrast to serum creatinine, which measures renal excretory function, NGAL is specifically induced in the damaged tubule and then released into the urine [15]. Only low levels of NGAL are detectable in the urine of healthy subjects [4]. Acute kidney injury leads to rapid NGAL mRNA upregulation in kidney tubules followed by marked increase in urine NGAL protein excretion [16]. More recently, urine NGAL has been shown to be elevated in patients with chronic tubulointerstitial disease [17, 18] and urine NGAL may be predictive of long term decline in renal function in nonproteinuric CKD, but limited data are available in glomerular diseases. Ding et al. found increases in urinary but not serum NGAL in patients with advanced IgA nephropathy levels consistent with local renal generation as the major source of urinary NGAL [19]. Hammad et al. found levels of urinary NGAL were higher in systemic lupus erythematosus patients with nephritis than those without nephritis [20]. Bolignano et al. showed that patients with membranous nephropathy had increased urine NGAL compared to controls [5]. Consistent with this, we found the levels of NGAL in glomerular diseases to be elevated by about 6-fold in glomerular diseases compared to normal subjects.

Proteinuria is an important direct mediator of tubular epithelial cell injury and is a strong predictor of renal disease progression [3]. Reducing proteinuria with immune modulating therapy or renin-angiotensin system blockers is the cornerstone of therapy for glomerular diseases [14]. Cross-sectional studies have shown that urinary NGAL increased in parallel with degree of proteinuria in glomerular diseases [5, 18, 19], but few studies have examined the changes of NGAL after treatment. In streptozotocin-diabetic mice, angiotensin receptor blockade which decreased proteinuria also lowered NGAL excretion [21]. Kuwabara et al. showed a reduction of NGAL in 4 nephrotic syndrome patients after treatment of proteinuria with immunosuppressive therapy [21]. In this study, we treated patients with biopsy-proven glomerular diseases according to standard guidelines [11] and found that the change in proteinuria strongly correlated with the change in NGAL excretion. Moreover, NGAL levels in patients with complete remission decreased to levels similar to healthy subjects. Our prospective data is consistent with a cross-sectional study in children with steroid-sensitive nephrotic syndrome in which subjects with active disease had higher NGAL than children in remission [18].

Several mechanisms may account for the strong correlation between proteinuria and urinary NGAL levels [22]. Passive loss of circulating NGAL through the damaged glomeruli could contribute to the increase in urinary NGAL level. Increased filtered albumin and other proteins could also overload the megalin-cubilin dependent reabsorption of NGAL in the proximal tubule leading to increased urinary NGAL excretion [21]. Excessive reabsorption of protein could result in direct tubular toxicity and increased synthesis of cytokines and complement activation leading to inflammatory cell infiltration, tubulointerstitial fibrosis, and subsequent nephron loss [2, 3]. Augmented production of NGAL may be a defensive compensatory response to prevent tubular cell apoptosis induced by proteinuria [23]. Increased NGAL production by damaged distal tubules might contribute to NGAL excretion in glomerular diseases [19]. Previous investigators found that NGAL excretion increased with the severity of chronic tubulointerstitial changes [19, 24]. A similar trend was observed in our study and would probably reach statistical significance if more patients with moderate to severe tubulointerstitial changes were included.

Acute tubular necrosis due to ischemia or nephrotoxins leads to a marked increase in NGAL excretion [4]. In nephrotic syndrome, low oncotic pressure can result in reduced renal perfusion and reversible acute tubular injury [25]. The levels of NGAL in patients with glomerular diseases were on average 10–100 times lower than levels in nonglomerular disease AKI controls. Despite clear differences in GFR, there were no overall differences in baseline NGAL levels between glomerular disease patients with histological features of tubular injury and those without, although a few patients with tubular injury had high NGAL levels in the same range as AKI controls. Correlations of urine NGAL and GFR have been observed in previous cross-sectional studies in CKD [5, 8, 26]. Lower numbers of patients with advanced disease and higher mean GFR in our subjects as well as the interfering effects of proteinuria could also account for the lack of relationship between NGAL and GFR. Taken together, this suggests that a combination of mechanisms likely contributes to the elevated NGAL excretion in glomerular disease [21]. Excess filtration of systemic NGAL and mal-processing in the proximal tubules appear to be dominant mechanisms given the strong correlations of NGAL with proteinuria although coexisting tubular injury might account for the very high levels of NGAL in some patients. GFR and chronic tubulointerstitial change appear to have less dominant roles.

Complete remission is a good predictor of long term prognosis in many types of glomerular diseases including lupus nephritis [27] and focal segmental glomerulosclerosis [28]. In our study, the baseline levels of NGAL in subjects with complete remission at follow-up were similar to those not in remission and the baseline NGAL level did not correlate with GFR at follow-up (*data not shown*). Thus NGAL level at baseline was not predictive of response to therapy but rather NGAL decreased with the resolution of proteinuria. Our results suggest that NGAL is not a useful biomarker for predicting therapeutic response in nondiabetic glomerular disease and raise cautions on the benefit of NGAL for predicting long term outcome in proteinuric CKD. Data showing predictive value of NGAL on outcome in GN is limited. A previous study found that high baseline NGAL was predictive of decline of renal function in membranous glomerular diseases [5]. In contrast to our subjects, many of these patients had low GFR at baseline and most subjects still had persistent proteinuria at follow-up. Residual proteinuria after therapy has been shown to be a strong predictor of adverse outcome in CKD [14]. In our study, patients with partial remission or resistant disease had no reduction of NGAL at follow-up. Future studies will be necessary to determine if the persistent elevation or progressive increase of NGAL during the course of therapy can serve as a useful prognostic marker of disease prognosis independent of residual proteinuria.

This study had several limitations which may have influenced the results. We included various types of glomerular diseases with varying severity of proteinuria and GFR. Patients were not given standardized regimen but were treated by individual physicians according to broad guidelines recommended by KDIGO. These factors may have influenced the numbers of patients achieving remission or the ability of baseline NGAL to predict outcome. However, this unselected group of patients is quite representative of patients with nondiabetic glomerular diseases in our nephrology practice and the results serve further to emphasize the importance of proteinuria on NGAL excretion, although we cannot determine the exact mechanism of this relationship. The follow-up period was quite short so we cannot fully evaluate the predictive value of baseline or posttreatment NGAL levels on long term outcome. This study is small in size so detailed disease specific differences cannot be evaluated.

## 5. Conclusions

In glomerular diseases, the prevailing level of proteinuria at baseline and at follow-up is a strong determinant of NGAL, whereas tubulointerstitial disease severity and GFR have lesser roles. Patients who achieve complete remission have greater reduction of urine NGAL with follow-up levels being similar to normal subjects. In contrast to the proposed benefit of NGAL in predicting long term outcome in nonproteinuric CKD, baseline NGAL levels may not be a useful biomarker to predict medium-term therapeutic response in proteinuric glomerular diseases with relatively preserved tubulointerstitium. Larger studies with longer follow-up involving patients with a broader spectrum of disease severity will be essential to determine if baseline or posttherapy levels of urine NGAL can provide additional prediction of long term outcome in proteinuric CKD beyond that of residual proteinuria.

## Supplementary Material

Baseline and follow-up laboratory parameters in patients with complete remission, partial remission and not in remission status at follow-up.

## Figures and Tables

**Table 1:** Baseline characteristics according to remission status at follow-up.

Baseline characteristics	All patients (*N* = 43)	By response to treatment		*p* value
		Complete remission (*N* = 10)	Not in remission (*N* = 33)	
Male, *n* (%)	15 (34.9%)	5 (55.6%)	10 (29.4%)	0.143
Age, years	45 ± 17	42 ± 17	47 ± 17	0.409
BMI, kg/m^2^	25.5 ± 3.9	26.7 ± 3.2	25 ± 4.1	0.19
Systolic BP, mmHg	135 ± 21	133 ± 12	137 ± 23	0.52
Diastolic BP, mmHg	79 ± 11	82 ± 7	79 ± 12	0.26
ACEI and/or ARB use	38 (88.3%)	8 (80%)	30 (90.9%)	0.52
Corticosteroids ± immunosuppressive agents (%)	25 (58%)	9 (90%)	16 (48.5%)	0.035^*∗*^
Albumin, g/dL	3.14 (0.59–3.88)	1.86 (0.78–3.88)	3.21 (0.59–3.88)	0.035^*∗*^
Cholesterol, mg/dL	250 (143–669)	338 (150–594)	240 (143–669)	0.060
Serum creatinine, mg/dL	1.21 (0.43–4.17)	1.21 (0.54–1.42)	1.21 (0.43–4.17)	0.141
Baseline GFR, mL/min/1.73 m^2^	66.2 (12.3–143.4)	77 (54–137)	59 (12–143)	0.09
Proteinuria, g/g creatinine	2.17 (0.09–9.23)	3.06 (0.11–9.23)	2.15 (0.09–9.15)	0.55

Data shown as mean ± SD or median (min–max). ^*∗*^ *p* < 0.05 considered significant.

ACEI, angiotensin converting enzyme inhibitor; ARB, angiotensin receptor blockade; BMI, body mass index; BP, blood pressure; GFR, glomerular filtration rates.
