# Supplementary material for: Effects of Therapy on Urine Neutrophil Gelatinase-Associated Lipocalin in Nondiabetic Glomerular Diseases with Proteinuria
Source: Int J Nephrol. 2016 Jul 25;2016:4904502. doi: 10.1155/2016/4904502 (PMC4976195; doi:10.1155/2016/4904502)
Supplement: Supplementary file 1 — Baseline and follow-up laboratory parameters in patients with complete remission, partial remission and not in remission status at follow-up. [file 4904502.f1.pdf]

Supplementary Table 1: Laboratory parameters according to remission status at follow-up

	Status at follow-up			P value	P value
Parameters	Complete Remission (n=10)	Partial Remission (n=16)	Resistant (n=17)	PR vs. Resist	PR vs. CR
$UPCR_1$	2.50 (0.54---9.23)	3.35 (1.45-9.15)	1.02 (0.54-6.60)	0.056	0.44
$UPCR_2$	0.15 (0.06-0.26)	0.80 (0.31-3.04)	1.62 (0.34-16.96)	**0.023**	**<0.001**
GFR_1	79 (54-137)	56 (12.3-143)	66 (25-143)	0.38	0.12
GFR_2	97 (71-130)	50 (12-130)	60 (12-140)	0.38	0.12
$NGAL_1$	29.3 (16.7-213.0)	24.6 (5.0-103.2)	23.2 (2.9-120.3)	0.86	1.0
$NGAL_2$	7.4 (1.6-66.0)	25.4 (2.9-139.2)	23.5 (0.5-359.7)	0.86	1.0
$\Delta UPCR_{2-1}$	-2.28 (-9.03- -0.48)	-2.12 (-6.44 --0.93)	0.01 (-1.78-14.1)	**<0.001**	**<0.001**
Δ GFR_{2-1}	+13.0 (-21.2-35.0)	-3.4 (-39.2-41.4	-2.6 (-26.5-71.7)	0.86	0.44
$\Delta NGAL_{2-1}$	-15.2 (-211.4-33.7)	2.2 (-77.3-115.8)	3.9 (-31.8-289.3)	0.86	**0.046**

Data as median (min-max).

Subscript 1 denotes baseline and subscript 2 denotes follow-up. Δ denotes change from baseline to follow-up (Follow-up minus baseline).

UPCR, Urine protein creatinine (g per g creatinine); GFR, glomerular filtration rate (ml/min/1.73m^2); NAGL, urine Neutrophil Gelatinase-Associated Lipocalin (ng/ml); CR, complete remission; PR, partial remission; Resist, Resistant
